# Quantitative Electroencephalography (QEEG) as an Innovative Diagnostic Tool in Mental Disorders

**DOI:** 10.3390/ijerph19042465

**Published:** 2022-02-21

**Authors:** Marta Kopańska, Danuta Ochojska, Agnieszka Dejnowicz-Velitchkov, Agnieszka Banaś-Ząbczyk

**Affiliations:** 1 Department of Pathophysiology, Institute of Medical Sciences, Medical College of Rzeszow University, 35-959 Rzeszow, Poland; 2 Department of Psychology, Institute of Pedagogy, College of Social Sciences, University of Rzeszow, 35-959 Rzeszow, Poland; dochojska@wp.pl; 3 ADEA, Biofeedback Center, 1000 Sofia, Bulgaria; adejnowicz@gmail.com; 4 Department of Biology, Institute of Medical Sciences, Medical College of Rzeszow University, 35-959 Rzeszow, Poland; agnieszkabanas@o2.pl

**Keywords:** generalized anxiety disorder, conditions, diagnosis, QEEG, EEG biofeedback

## Abstract

Quantitative electroencephalography (QEEG) is becoming an increasingly common method of diagnosing neurological disorders and, following the recommendations of The American Academy of Neurology (AAN) and the American Clinical Neurophysiology Society (ACNS), it can be used as a complementary method in the diagnosis of epilepsy, vascular diseases, dementia, and encephalopathy. However, few studies are confirming the importance of QEEG in the diagnosis of mental disorders and changes occurring as a result of therapy; hence, there is a need for analyses in this area. The aim of the study is analysis of the usefulness of QEEG in the diagnosis of people with generalized anxiety disorders. Our research takes the form of case studies. The paper presents an in-depth analysis of the QEEG results of five recently studied people with a psychiatric diagnosis: generalized anxiety disorder. The results show specific pattern amplitudes at C3 and C4. In all of the examined patients, two dependencies are repeated: low contribution of the sensorimotor rhythm (SMR) wave amplitudes and high beta2 wave amplitudes, higher or equal to the alpha amplitudes. The QEEG study provides important information about the specificity of brain waves of people with generalized anxiety disorder; therefore, it enables the preliminary and quick diagnosis of dysfunction. It is also possible to monitor changes due to QEEG, occurring as a result of psychotherapy, pharmacological therapy and EEG-biofeedback.

## 1. Introduction

Fear accompanies us throughout our lives. It has its advantages, namely, it makes us more vigilant in various difficult situations, and therefore protects against threats. It also mobilizes specific adaptation actions. Unfortunately, if anxiety is chronic and severe, it can disorganize behavior, “paralyze” and hinder everyday functioning. As a result of the stressors at work, difficult situations that are not easy to deal with, various types of anxiety symptoms may appear in the form of phobias, panic attacks, post-traumatic stress disorder, or generalized anxiety disorder (GAD). An anxiety has no specific cause; it is a subjective phenomenon that triggers a sense of threat, and it can refer to imaginary situations where the individual anticipates what may happen and is afraid of certain situations that are not justified. Additionally, the anxiety is usually caused by unconscious factors. Thus, people often become helpless in the face of their own anxiety. The method of analyzing objectively functioning factors depends on one’s subjective interpretation resulting from individual predispositions, largely related to the interaction of biological factors and psychosocial experiences of a given individual [1]. Taking into account the latest ICD-11 classification, various types of anxiety symptoms were included in the general group called “anxiety and fear disorders” [2,3].

Analyses carried out by various researchers indicate that these disorders are the result of the interaction of biological and psychosocial factors (e.g., family and school environment, further environment and related crises, difficult experiences) [4,5,6,7].

Particularly strong, long-term stressors can increase anxiety. As research shows, unfortunately, the long-lasting coronavirus pandemic has also increased the level of anxiety in many people [8,9].

On the one hand, anxiety is the source of many mental disorders, and on the other hand, it is their symptom. Particularly severe and long-lasting anxiety is associated with generalized anxiety disorder. Generalized anxiety disorder is one of the most common mental disorders. In the case of generalized anxiety disorder, we deal with chronic anxiety manifested in the form of a constant feeling of anxiety and worry in connection with an unclear sense of threat in the absence of certain external factors. The main accompanying symptoms include problems with sleep, concentration, fatigue, excessive muscle tension, as well as chronic vegetative symptoms (e.g., headache, gastric ailments, e.g., epigastric pain, problems with swallowing, obstruction, tachycardia) [10,11].

There are also panic attacks that are not as strong as in panic disorder [12]. However, the dominant symptom is excessive worrying [13]. The consequence of long-term, severe anxiety are difficulties in controlling one’s emotional states, as well as serious dysregulation of behavior in various spheres of life, which leads to problems at work and in relations with the closest family members and social environment. Strong anxiety can even lead to an inability to carry out the simplest activities of daily living. Constant concentration on oneself and one’s mental state, as well as the feeling of suffering, is associated with neglecting loved ones and giving up various needs.

In the differential diagnosis, it is necessary to exclude thyroid diseases and other somatic diseases, which are also accompanied by a state of tension and various symptoms of anxiety [14]. Diagnosis requires severe symptoms over an extended period of time where the person is unable to cope with and control the symptoms. “Statistically, the average incidence throughout life is approx. 5%” [15]. Generalized anxiety disorder may coexist with other mental disorders: dysthymia, PTSD (Post-traumatic Stress Disorder), panic attacks, social phobia, depression. Moreover, the abuse of alcohol and psychoactive substances is often associated with increased anxiety [16].

Correct diagnosis of anxiety disorders is the basis for appropriate therapy. Usually, generalized anxiety disorder requires long-term treatment, and the intense symptoms often last from a few to several years. Scientific research shows that, among sick people, the percentage of patients in remission after one year is 15%, and after two years 25%. On the other hand, after five years, approximately 38% of patients are in remission. Analyzing the frequency of recurrence of symptoms, it was found that after a year, about 10% of people experience a relapse, and after three years this percentage increases to about 25% [16]. Both biological and environmental conditions of the disorder should be taken into account in the diagnosis process. Taking into account biological factors, researchers focus on the important role of neurotransmitters (including dopamine, norepinephrine, and serotonin), as well as an increased level of activation of specific areas of the brain. The neurophysiological basis for the relationship between the appearance of unexplained severe anxiety symptoms and the declarative memory related to the hippocampus and emotional memory based on the activity of the amygdala [17] was found. The importance of biochemical imbalance in the body as a result of vitamin B and C deficiency and infections is also noted. This disorder occurs in both adults (about 20%) and children (5.7% to 12.8% under 18 years of age). The incidence of GAD depends on gender (twice as high in women as in men) [18,19,20]. Anxiety disorders are also the result of negative experiences. Often, as a result of the occurrence of certain situations that evoke association with an unpleasant, stress-inducing experience, anxiety is activated [19]. Understanding the patient’s own experiences facilitates self-control, which in turn may have an impact on reducing the level of anxiety. On the other hand, it is necessary to modify one’s behavior to effectively cope with difficulties.

## 2. Assessment of Progress in the Treatment of People with Generalized Anxiety Disorder with the Use of QEEG

Due to various, overlapping determinants of anxiety disorders, psychotherapy is used for people with this type of symptoms, often combined with pharmacotherapy [20]. Taking into account the treatment standards [21], benzodiazepines, for example, alprazolam, are most often used temporarily, because this group of drugs reduces anxiety symptoms in a short time [22]. The rapid action of these types of drugs also seems to be important in connection with the patient’s involvement in this process. It is important that the patient does not give up treatment with a feeling of ineffectiveness. Medications are needed when emergency intervention is needed, but also to help manage anxiety. However, the enormous risk of becoming addicted to benzodiazepines has to be taken into account.

Usually, a person, due to the presence of severe anxiety, is not able to define the specific reasons for his/her frame of mind. As a part of psychotherapy, the awareness of the factors and events that cause these ailments often makes it easier to understand what the person is going through, and gives the possibility of partial control over the situation. However, it is also important to work through difficulties and modify behaviors in the process of psychotherapy. The psychodynamic approach is practiced frequently here; however, cognitive-behavioral therapy (CBT) is more and more often considered the most effective method [23,24]. Taking into account the psychoanalytical approach, the source of fear is unconscious, intrapsychic conflicts. Cognitive-behavioral therapy, in turn, focuses on identifying non-functional/irrational beliefs that cause fear and their transformation. It is important here to practice (among others) self-control skills of anxiety-triggering symptoms and imaginative exposure of anxiety triggers, as well as to work on the control of worrying and the use of relaxation techniques that reduce tension [25].

Taking into account the biomedical methods of diagnosing generalized anxiety disorder, it is important to carry out a differential diagnosis that excludes the existence of other diseases. In addition to a detailed interview with the patient, psychological tests and laboratory tests (e.g., analysis of pathogens or deficiencies of specific substances), molecular genetics or brain neuroimaging, including magnetic resonance and computed tomography, can be used. The importance of EEG (electroencephalography) and neurofeedback in the diagnosis and treatment of anxiety disorders is increasingly emphasized in the literature [26]. Neurofeedback, also called neurotherapy, is a type of biofeedback that presents real-time feedback from brain activity in order to reinforce healthy brain function through operant conditioning. QEEG and neurofeedback are methods that can be useful to evaluate the progress in the treatment of patients with this type of dysfunction and in therapy. It is a non-invasive, easy-to-use, and economical method.

QEEG (quantitive electroencephalogram) is a quantitative analysis of the EEG record, where the data are digitally coded and subjected to statistical analysis using the Fourier transform algorithm [27].

Depending on various mental states, we can talk about a different record of brain waves of different frequencies. Taking into account the types of brain waves, the following should be mentioned: delta waves (they are typical during deep sleep—without dreams); theta waves, dominant in the state of emotional agitation, the creative process, the activity of the imagination, as well as in the time of rest after exercise; alpha waves are generated in a state of rest, relaxation and meditation. If their frequency is too high, they indicate difficulties in concentration, and if they are too low, it may indicate a high body tension, stress, and insomnia; beta waves occur in a situation of concentration and are associated with high activity of neurons (however, their too high intensity is an indicator of anxiety and stress); gamma waves indicate cognitive activity and dominate during the REM phase of sleep [28].

In addition, the amplitudes of fast waves, according to the standards, should be up to 50% higher in a given dominant hemisphere, and in the non-dominant hemisphere, higher amplitudes are expected for slow waves—delta, theta, alpha, and SMR. According to the neurotherapeutic practice, however, a small correction of these percentages can be introduced, taking into account the contributions of alpha and SMR waves. Namely, under the influence of emotional tension, these waves tend to increase, which may result from difficulties in subcortical inhibition. The planning of neurofeedback training is about amplifying these waves in the range: alpha up to 16% and SMR up to 12%. In turn, 3% of the contribution of these waves should be left in the activity of beta1 and beta2 waves [29,30,31].

Thus, the amplitude of delta waves should be greater than the amplitudes of theta waves, the amplitude of theta waves greater than the amplitudes of alpha waves, the amplitude of alpha waves greater than the amplitudes of SMR waves, and the amplitude of SMR greater or equal to the beta1 and beta2 waves. On the other hand, the amplitudes of SMR waves should not be lower to half (1/2) of the amplitudes of the theta waves, and the amplitude of alpha waves lower than the amplitude of the theta waves and higher than the amplitude of the SMR waves, and always higher or equal to the ratio of the amplitudes of the beta2 waves [32].

EEG-biofeedback (neurofeedback) training is developed based on a detailed analysis of diagnostic reports of quantitative EEG records. The purpose of EEG Biofeedback is to design interactions in such a way as to shape brain waves by reducing or increasing the share of a specific wave. The exercises are designed to teach the patient what state of the brain is optimal for its proper functioning and what situational factors increase the efficiency of the brain. QEEG allows one to detect deviations in the function of the brain as a result of the action of specific stimuli and at the same time allows for checking in what circumstances brain waves return to normal [32]. Through the systematic repetition of QEEG tests, changes in the brain can be evaluated, which is useful for assessing changes in the brain and the effectiveness of therapy.

QEEG is most often used to study brain changes related to Alzheimer’s, Parkinson’s, migraines, and epilepsy [33,34,35,36,37]. An increasing number of researchers find this method useful in the diagnosis of ADHD [38,39] and various mental dysfunctions, including depressive and schizophrenic syndromes [40,41,42] and addictions [43].

The correct interpretation of the results requires obtaining additional information about the patient, including symptoms reported by him and medications taken. The patient’s age should also be taken into account, as changes occur during human life resulting from maturation, brain development, and dementia processes [28,44]. The QEEG studies conducted so far confirm the similarity of brain waves in people with ADHD and bipolar disorder [45]. Thanks to the diagnosis of QEEG, having the image of brain waves, it is possible to develop EEG-biofeedback (neurofeedback) therapy personalized to the individual patient [40].

The research conducted by Ribas et al. [46] found an association between the symptoms of anxiety and the category of hot temporal lobes (T3 and T4) of the TLC technique using the TQ-7 method. The activation of the temporal lobes, T3 and T4, seems to indicate an excessive activity of the amygdalae [47].

## 3. Material and Method

Quantitative electroencephalography (QEEG) data were collected by a researcher with Board Certification in Neurofeedback. Taking into account the basic principles of QEEG analysis in an adult (at rest and with eyes open), it is assumed that the lower the frequency of the waves, the lower the amplitude (delta less than 20 mV, theta in adults less than 15 mV, alpha in adults less than 10 mV, SMR, beta1 and beta2 within 4–10 mV). The QEEG data were collected by measuring all waves from central points (Cz, C3, C4), based on the international 10–20 system [48]. The EEG signal was transformed using Cz montage (Cz is the common reference site) [48,49,50] and by quantifying with the Elmiko, DigiTrack software (version 14, PL) (ELMIKO, Warsaw, Poland) to examine the central waves asymmetry. The QEEG results of five patients with a psychiatric diagnosis of Generalized Anxiety Disorder (in accordance with criteria International Classification of Disease ICD-10) are presented below. The study performed included delta, theta, alpha, SMR, beta1, and beta2 waves at electrodes Fz, Cz, C3, C4, P3, P4, F3, F4. Due to the desire to present significant results, we took into account the electrodes from the central lane, in which there were significant changes in the most important waves for GAD.

## 4. Analysis of the QEEG Results of Patients with a Psychiatric Diagnosis of Generalized Anxiety Disorder

For comparison and analysis, we present the results of five recently admitted patients with generalized anxiety disorder: AD—female 47 years old, GG—female 45 years old, SK—female 50 years old, TD—female 48 years old, and GP—male 33 years old. A brief presentation of the patients with GAD is provided in the Table 1.

## 5. The Results of the QEEG Study of AD Women—47 Years

In the examined AD woman, delta with an amplitude of 8.73 mV in C3 and 8.42 mV in C4 was found and it is normal. The difference in amplitudes between the left and right hemispheres is also correct, as it does not exceed 20%. On the other hand, theta in C3 has a value of 9.62 mV and in C4 9.53 mV, so it is also normal, but higher than delta. We consider the contributions of theta amplitudes higher than the delta amplitudes to be incorrect. The amplitudes on alpha in C3—7.94 mV and C4—11.1 mV exceed the normal difference of 20%, which we take as an abnormal indicator, and at the same time reveal the activation of the limbic system in the right hemisphere. The alpha amplitude is also higher than the theta amplitude, which also indicates an abnormality. The alpha amplitude itself in the left hemisphere is normal up to 10 mV, while it is higher (above normal) in the right hemisphere. The SMR amplitudes in C3 are 5.75 mV and C4—6.62 mV, and the amplitude difference is 20%, so it is correct. The amplitudes of the beta1 and beta2 waves are much higher concerning the amplitudes of the SMR wave. Beta1 increases by 2.5–3 mV and amounts to, respectively, in C3—8.95 mV and C4—8.05 mV, although it remains within the normal range. The amplitudes of beta2 waves increase by another 1.5–3 mV, in C3 10.27 mV, and C4 11.39 mV, and these slightly exceed the norm of 10 mV. We observe greater beta1 amplitude in C3 than alpha amplitudes and in both hemispheres greater proportions of beta2 amplitudes concerning alpha, theta, and even delta amplitudes.

**Figure 1:**
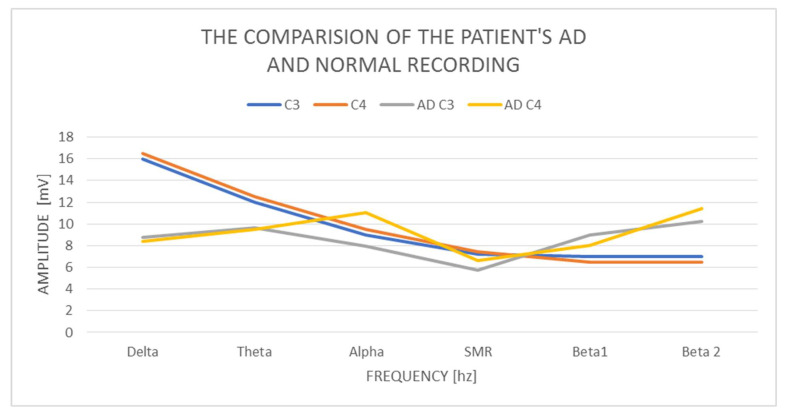
The comparison of the patient’s AD and normal recording.

Examination of the AD patient shows low contributions of delta wave amplitudes concerning other waves (Figure 1). There is a large left–right asymmetry in the amplitudes of the alpha wave. We also observe a very large increase in the amplitudes of beta1 and beta2 waves in both hemispheres, and beta2 exceeds the amplitudes not only of alpha but also of theta and delta. In a calm activity model, the amplitudes of beta waves should be no greater than ½ theta.

## 6. The Results of the QEEG Study of the GG Woman—45 Years of Age

In GG, women aged 45, the delta amplitude in the C3 study is 10.15 mV and in C4 9.96 mV, and its power is normal. The difference in amplitudes between the left and right hemispheres is also correct and does not exceed the permissible difference of up to 20%. The amplitude of the theta wave in C3 is 8.53 mV and in C4 7.81 mV; it is normal and does not exceed the delta wave amplitudes. The amplitudes of the alpha waves in C3—8.72 mV and C4—9.03 mV—in both hemispheres—are higher than the amplitudes of theta, which is an abnormality. The SMR amplitudes in C3 are 6.71 mV and in C4—6.50 mV, and the difference in amplitudes is 20%. The amplitudes of the beta1 and beta2 waves increase significantly concerning the amplitudes of the SMR wave. Beta1 increases in C3 to 8.86 mV and C4—7.17 mV. The amplitudes of the beta2 waves also increase and are in C3 8.52 mV and in C4 8.17 mV. 

**Figure 2:**
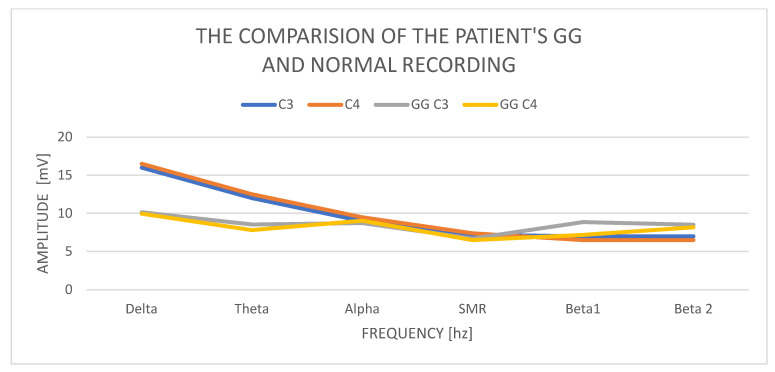
The comparison of the patient’s GG normal recording.

Examination of the GG patient shows low contributions of delta wave amplitudes in relation to other waves, especially the beta1 and beta2 wave amplitudes (Figure 2). The left–right asymmetry is visible in the beta1 wave amplitudes and higher in the left hemisphere. We also observe the alignment of the amplitudes of the beta1 and beta2 waves with the amplitudes of the alpha waves. In a state of psychophysical calm, the amplitudes of beta waves should not exceed ½ theta. Equalized amounts of beta wave amplitudes with alpha wave amplitudes always indicate anxiety and stress activity.

## 7. The Results of the QEEG Study of the Female SK—50 Years

A study of a 50-year-old female SK patient indicates that the delta amplitude in the study is C3 10.46 mV and C4 8.99 mV. So, the amplitude is correct. The difference in amplitudes between the left and right hemispheres is also correct and does not exceed the normal difference of up to 20%. The amplitude of the theta wave in C3 is 7.26 mV and in C4—6.21 mV, so it is also normal and does not exceed the amplitudes of the delta waves, but in general it is low amplitude. In turn, the amplitudes of alpha waves in C3—7.53 mV and C4—7.21 mV—in both hemispheres—are higher than the amplitudes of theta, similar to the previous cases described above, which is an abnormality. The SMR amplitudes in C3 are 5.86 mV and in C4—8.57 mV and the difference in amplitudes exceeds 20% of the norm. The amplitudes of the beta1 and beta2 waves increase in relation to the amplitudes of the SMR wave. Beta1 increases in C3 to 6.61 mV and C4 significantly to—10.61 mV. The amplitudes of the beta2 waves also increase, and there are visible differences between the hemispheres and they are in C3 11.38 mV and C4 19.75 mV. The beta2 wave amplitudes indicate a very large left–right hemisphere asymmetry—almost 100%—and in both hemispheres they are outside the norm of 10 mV.

**Figure 3:**
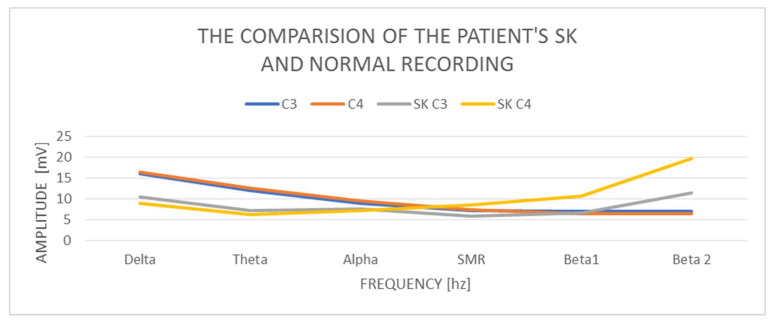
The comparison of the patient’s SK and normal recording.

The examination of the SK patient shows low proportions of the delta wave amplitudes in relation to the other waves, especially the beta1 and beta2 wave amplitudes (Figure 3). There are visible left–right asymmetries in the beta1 and beta2 wave amplitudes, with a marked increase in the right hemisphere. We also observe the amplitudes of the beta1 and beta2 waves, which are higher than the amplitudes of the alpha waves, which is an abnormality.

## 8. The Results of the QEEG Study of the TD Woman—48 Years

In the study of a 48-year-old woman TD, we obtain delta with an amplitude of C3—11.96 mV and C4—11.60 mV. Therefore, we judge it as correct in relation to the norms. The theta in C3 has a value of 9.81 mV and in C4—9.21 mV, so it is normal and has the correct relationship to delta amplitudes. The amplitudes of the alpha waves in C3 are 5.87 mV and in C4—5.39 mV, and they clearly decrease in relation to the amplitudes of theta waves. The asymmetry in the alpha wave is normal, but we notice increased amplitudes of the slow waves (delta, theta, and alpha) in the left hemisphere. The amplitudes of the SMR wave in C3 are 3.51 mV and in C4—3.27 mV, the difference in amplitudes is 20%, but the amplitude itself is lower than the norm. The amplitudes of the beta1 and beta2 waves increase significantly in relation to the amplitudes of the SMR wave. The beta1 amplitude increases in C3 to 4.73 mV and in C4—4.30 mV. The tendency to increase the amplitudes of cortical waves is also observed in beta 2: in C3—7.2 mV and in C4—6.06 mV. The amplitudes of the beta1 and beta2 waves are within the normal range (4–10 mV), but in relation to the other low wave amplitudes, their influence is increased. The amplitudes of all cortical waves are higher in C3, as are the low-frequency waves.

**Figure 4:**
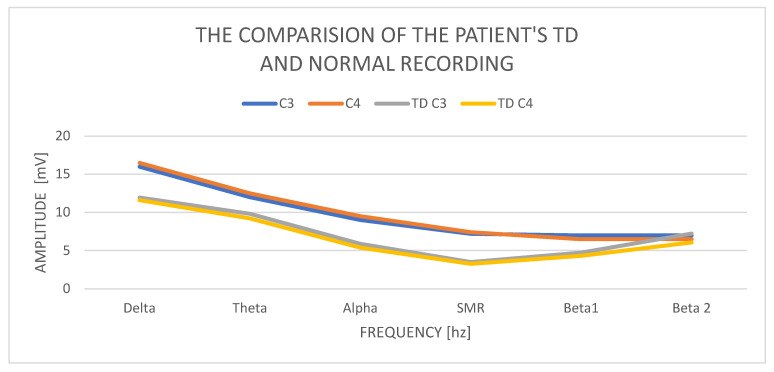
The comparison of the patient’s TD and normal recording.

Examination of the TD patient shows low influence of all wave amplitudes (Figure 4). There is a tendency to increase the amplitudes of the beta1 and beta2 waves, with a slight increase in the left hemisphere.

## 9. The Results of the Male’s QEEG GP Study—33 Years

In a study of a 33-year-old GP male, we obtain a delta waves with an amplitude of C3—10.93 mV and C4—8.87 mV. Amplitude contributions in C3 are 11.59 mV and C4—10.21 mV, so they are normal but higher than delta. The alpha amplitudes in C3 are 6.85 mV and C4 amplitudes 6.45 mV. The SMR amplitudes in C3 are 4.86 mV and in C4—4.24 mV and the difference in amplitudes is 20%, which is normal. The amplitudes of beta1 waves are, respectively, in C3—4.51 mV and C4—4.24 mV. Beta2 wave amplitudes increase and are at C3—6.63 mV and in C4—5.75 mV.

**Figure 5:**
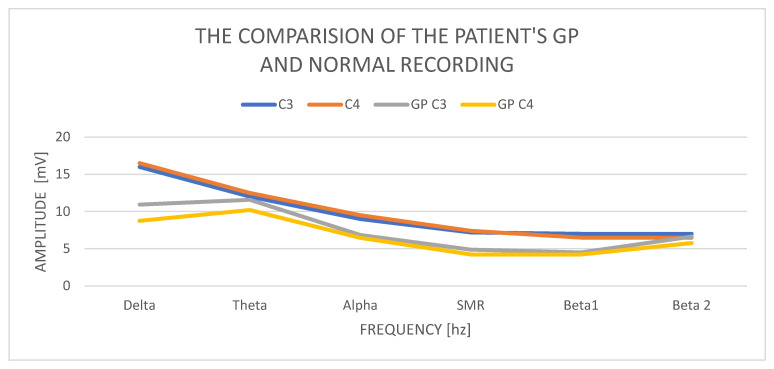
The comparison of the patient’s GP and normal recording.

Examination of a GP patient shows low contributions of all wave amplitudes (Figure 5 (comparison of the GP recording with a norm)). We observe two basic abnormalities: an increase in the amplitudes of the theta waves in relation to the amplitudes of the delta waves and an increase in the amplitudes of beta2 in both hemispheres, with a slight increase in the left hemisphere.

## 10. Discussion

The comparison of the patients’ results with the diagnosis of generalized anxiety disorder allows for the conclusion that in some respects they are similar. On the other hand, we can talk about individual differences between each person.

**Figure 6:**
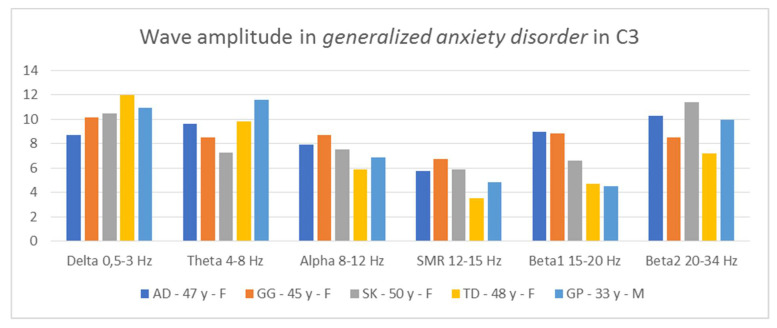
Wave amplitude in patients diagnosed with generalized anxiety disorder in C3.

**Figure 7:**
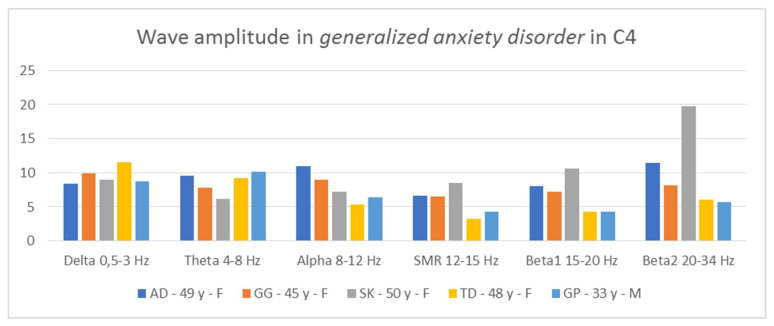
Wave amplitude in patients diagnosed with generalized anxiety disorder in lead C4.

Thus, it is possible to indicate general tendencies in the field of wave amplitudes in people diagnosed with generalized anxiety disorder. The comparison of the amplitudes in C3 and C4 (Figure 6 and Figure 7) allows for the conclusion that in all patients two relationships are repeated: low contributions of SMR wave amplitudes and high beta2 wave amplitudes, higher or equal to the alpha amplitudes.

In three AD, SK, and TD patients, the amplitudes of the beta2 waves are greater than those of the alpha waves. In two patients, AD and GP, we see greater proportions of theta in relation to delta in both hemispheres. In turn, in two people, GG and SK, we notice greater contributions of alpha waves in relation to theta in both hemispheres. In four patients, we observe large asymmetry between the hemispheres, but they concern different waves. In the AD test, the asymmetry concerns the amplitudes of the alpha waves, in the GG test—the beta1 amplitudes, SK—the beta1 and beta2 amplitudes, and the GP test—the delta amplitudes. Thus, in the studied patients, there are common features regarding wave amplitudes, which may be useful in differentiating disorders.

From QEEG records of people with attention deficit and hyperactivity disorder, tested according to the same procedures [39], it emerges that, in this group of people, the following relationships were found: high amplitudes of low-frequency waves—delta, theta, and alpha—and low amplitudes of high-frequency waves in relation to low-frequency waves. The amplitudes of high-frequency waves were comparable—SMR, beta1, and beta2. The amplitudes of beta2 waves were lower than those of alpha and theta. Thus, the brain wave image of people with generalized anxiety disorder differs from the results specific for people with ADHD, although each of these groups of respondents is characterized by an increased level of anxiety.

In turn, according to the research of Heller et al. [48], people with high levels of anxiety have an increased pattern of alpha activity in the right frontal lobe of the brain (segment) compared to people with low levels of anxiety. Similarly, higher alpha activity was found in people displaying social phobia and panic attacks [49,50,51,52,53,54].

Research limitations:

1. Sample size.
2. Lack of a comprehensive research base in the research area.

Research strengths:

1. Qualitative research.
2. Timely study topics. The study may suggest the approach of future researchers.

## 11. Conclusions

In conclusion, the obtained results indicate that QEEG is a valuable tool for the diagnosis of the specificity of brain waves in people with generalized anxiety disorder. However, it is necessary to carry out this type of diagnosis in a wider population of people with this type of disorder. Our analyses are an introduction to further research. It is important to compare the obtained results with the results of people revealing abnormalities in psychosocial functioning, which are based on neurological factors.

A QEEG diagnosis is a quick way to determine if there are any abnormalities. In addition, it allows one to learn about some of the mechanisms of the disease.

Moreover, based on multiple QEEG tests at intervals, it is possible to obtain a picture of possible changes, e.g., in the context of pharmacological therapy, psychotherapy, and EEG biofeedback. Based on the measurements of the amplitude of the rhythms based on the DigiTrack system, a change protocol is created and the range of impacts is planned, shaped to the individual needs of a given person. Through systematic, specific types of biofeedback exercises, the waves produced in the brain are changed. So, it is recommended that our investigation into this line of research in patients with anxiety disorder is continued, as the QEEG can serve not only for diagnosis but also to improve their quality of life.

## Figures and Tables

**Table 1:** Patients with GAD.

Patient	Sex	Age (Years)	Education	Profession	Medical History
AD	Female	47	higher	economist	GAD without any others disorders
GG	Female	45	higher	credit advisor	GAD without any others disorders
SK	Female	50	higher	paperwork	GAD without any others disorders
TD	Female	48	higher	paperwork	GAD without any others disorders
GP	Male	33	higher	economist	GAD without any others disorders

## Data Availability

The datasets generated during and/or analyzed during the current study are available from the corresponding author on reasonable request. Compliance with Ethical Standards Conflict of Interest on behalf of all authors.
